# Supplementary figures and images for: ZEB1 insufficiency causes corneal endothelial cell state transition and altered cellular processing
Source: PLoS One. 2019 Jun 13;14(6):e0218279. doi: 10.1371/journal.pone.0218279 (PMC6564028; doi:10.1371/journal.pone.0218279)

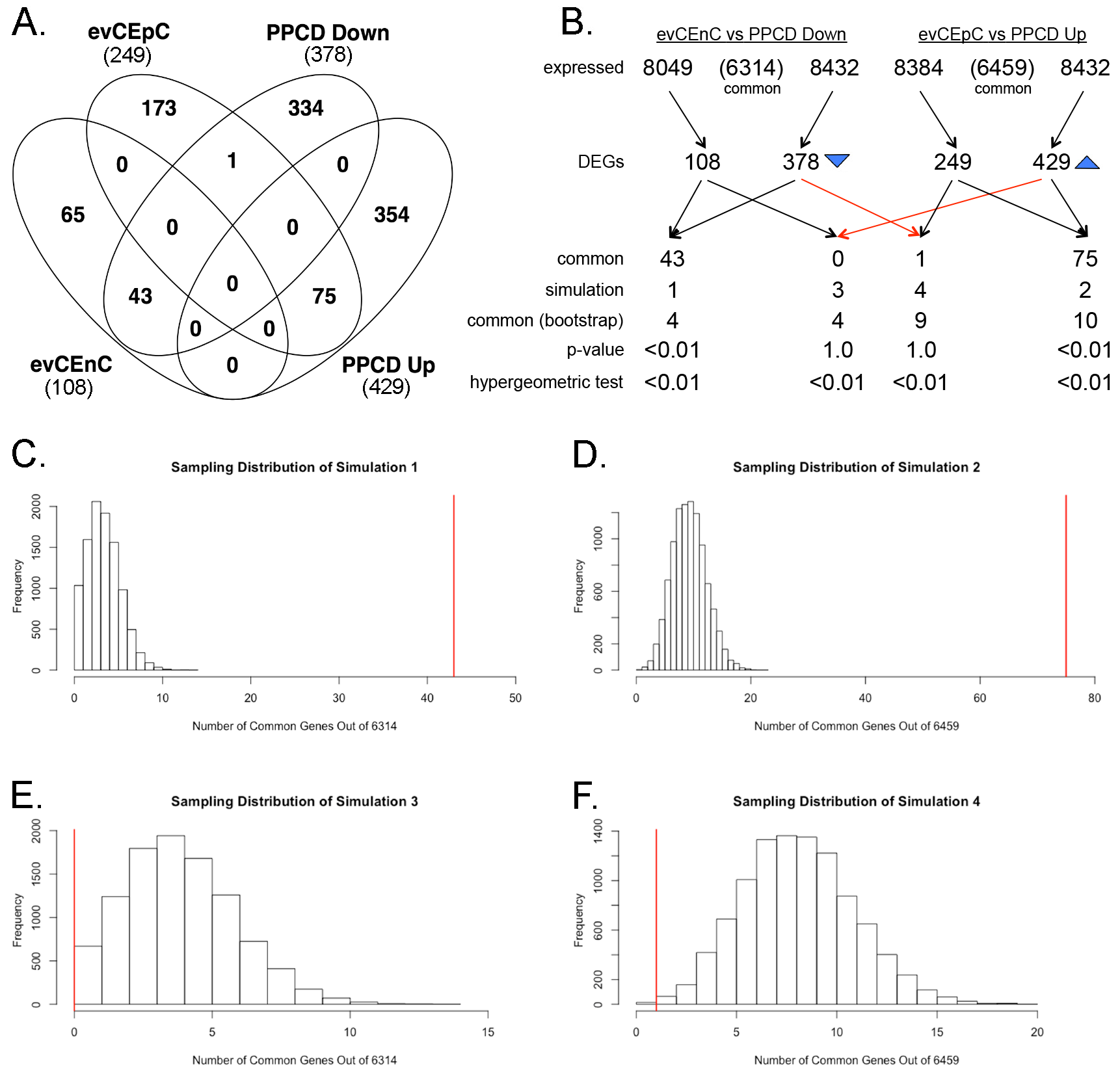

Supplement: S1 Fig — (A) Venn diagram comparing evCEpC- and evCEnC-specific genes with genes differentially expressed in PPCD. Seventy-six evCEpC-specific genes were differentially expressed in PPCD endothelium; 75 (99%) demonstrated increased expression. Forty-three evCEnC-specific genes were differentially expressed in PPCD endothelium; 43 (100%) demonstrated decreased expression. (B) Flowchart of number of genes used for statistical testing using a bootstrap approach and summary of results of 10,000 simulations for each scenario. The results of the hypergeometric test (hgt) are also included. Blue arrowheads indicate direction of differential expression. (C) Sampling distribution of scenario 1 where on average 4 genes were expected by chance to be both downregulated in PPCD and evCEnC-specific. Red line indicates observed value (43), which deviates significantly from the mean of the distribution and is not expected by chance alone (p<<0.01; hgt p<0.01). (D) Sampling distribution of scenario 2 where on average 10 genes were expected by chance to be both upregulated in PPCD and evCEpC-specific. Red line indicates observed value (75), which deviates significantly from the mean of the distribution and is not expected by chance alone (p<<0.01; hgt p<0.01)). (E) Sampling distribution of scenario 3 where on average 4 genes were expected by chance to be both upregulated in PPCD and evCEnC-specific. Red line indicates observed value (3), which deviates significantly from the mean of the distribution (p = 1.0; hgt p<0.01), and is not expected by chance alone. (F) Sampling distribution of scenario 4 where on average 9 genes were expected by chance to be both downregulated in PPCD and evCEpC-specific. Red line indicates observed value (1), which deviates significantly (p = 1.0; hgt p<0.01) from the mean of the distribution, and is not expected by chance alone. (TIF) [file pone.0218279.s001.tif]

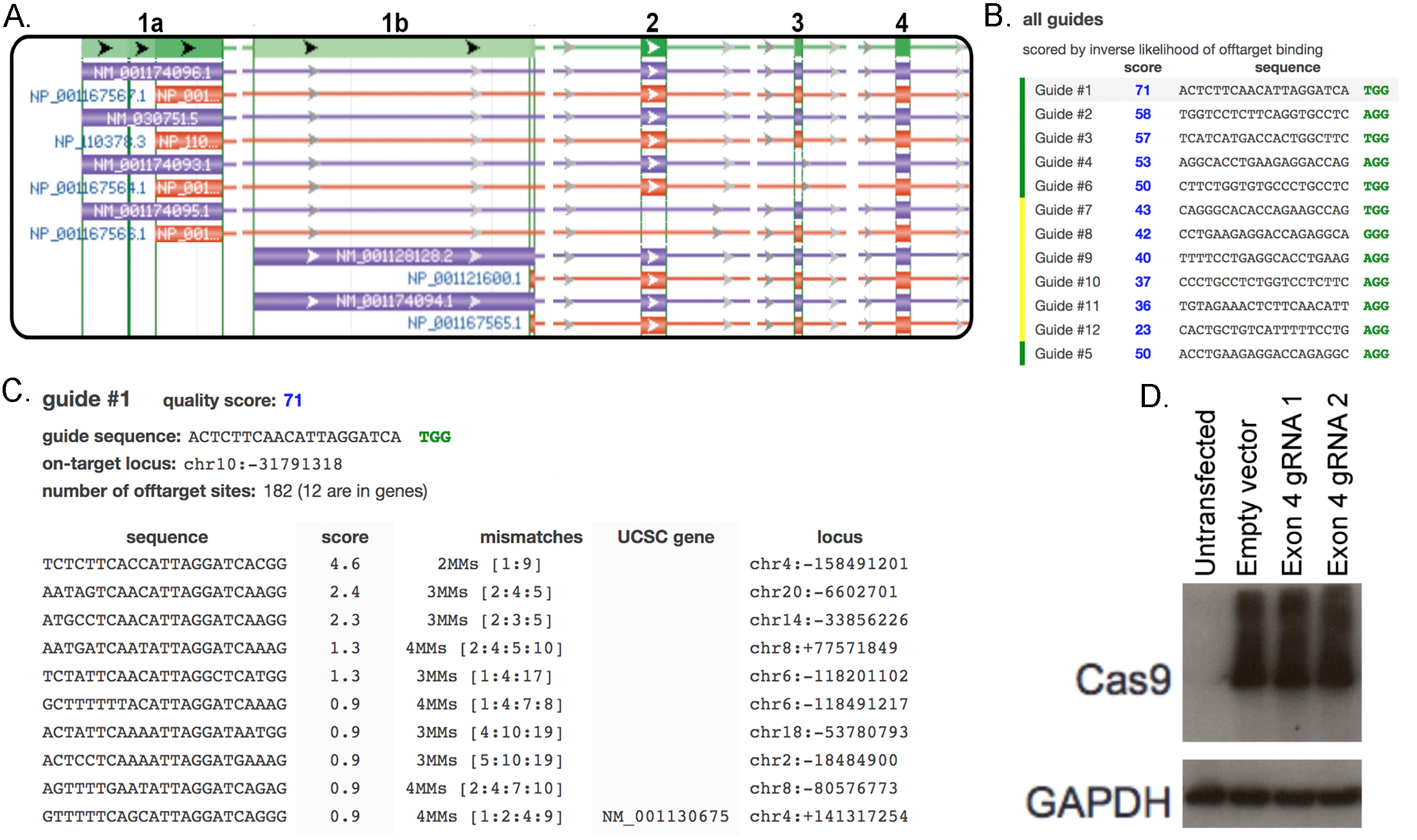

Supplement: S2 Fig — (A) Screen capture image showing annotated ZEB1 transcript variants present in the GRCh37.13/hg19 genome build. This build was used because the crispr.MIT.edu guide RNA design tool also utilized the hg19 genome build. Exon 4 was the earliest exon that was present in all ZEB1 transcript variants and protein isoforms. Exons are indicated by broad colored lines, which are joined by intronic sequences indicated by thin colored lines. Image was modified to accommodate presentation in this figure. Gaps in lines represent intronic sequence that was removed. Exons 5–9 are not shown. (B) List of guides designed to target exon 4 in ZEB1. Guides were ranked by score (blue font), which accounts for both on-target and off-target activity. The guide with the highest score (Guide #1) was used for CRISPR-Cas9-mediated editing of exon 4 in ZEB1. The PAM sequence is indicated by green font. (C) The top ten potential off-target sites for guide #1. Several parameters are accounted for in scoring off-target sites, and include number of mismatches, mismatch position and mean pairwise distance between mismatches. Sanger sequencing was used to screen these potential off-target sites (see S5 Fig). (D) Western blot demonstrating the presence of Cas9 protein in CEnC whole-cell lysates after transfection with gRNA/Cas9 DNA construct. GAPDH was used as a loading control. (TIF) [file pone.0218279.s002.tif]

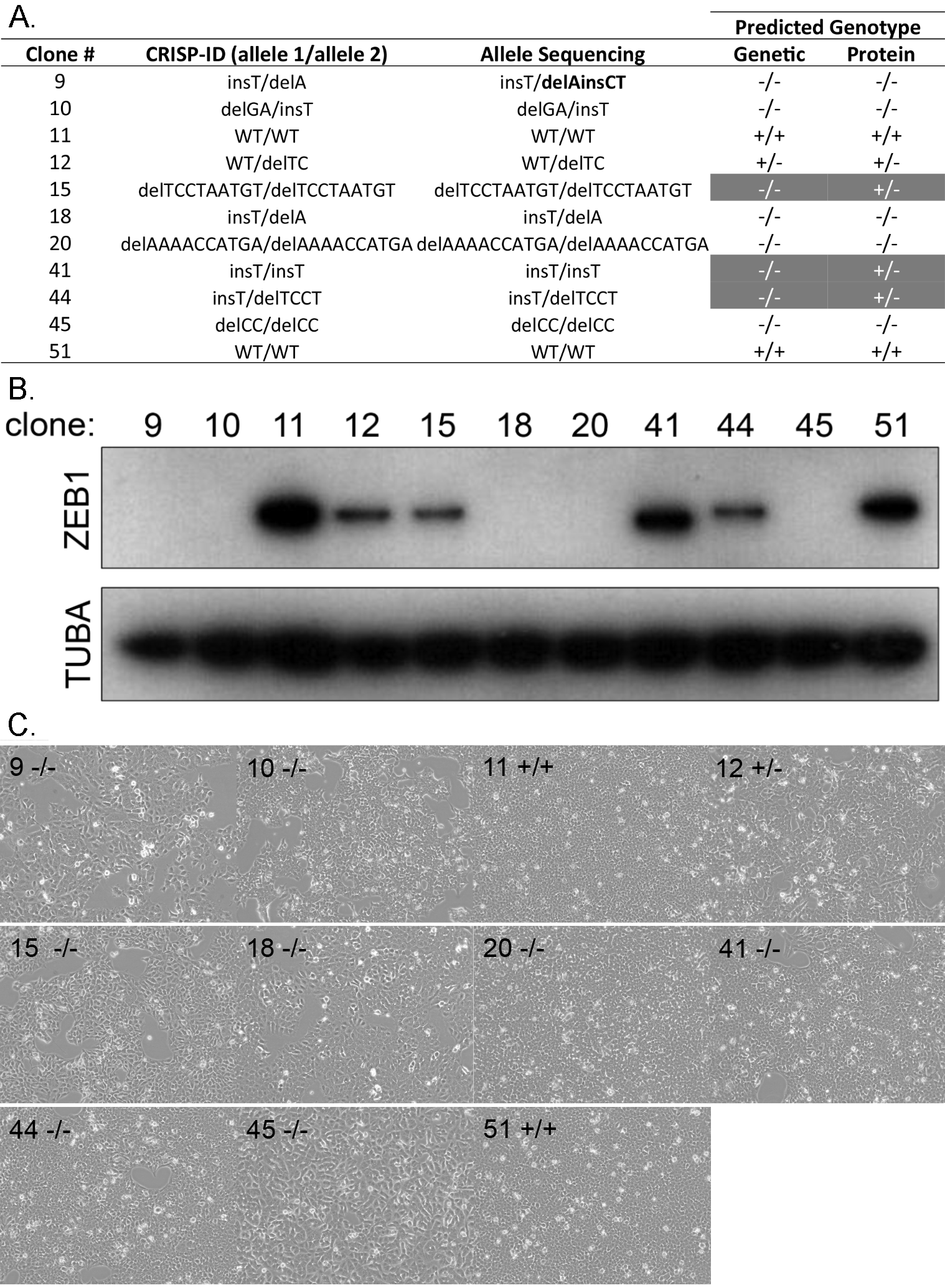

Supplement: S3 Fig — (A) List of selected clones on which additional genetic and molecular characterization was performed. Sanger sequencing of exon 4 of each of the selected clones was performed and the sequencing traces were analyzed using CRISP-ID, which predicted the indels that were introduced after Cas9-mediated DNA cleavage and NHEJ repair. Allele-specific sequencing was performed to validate the indels predicted by CRISP-ID. A simplified description, using “-”for indel and “+” for wild type, of the predicted genotype was compiled for each the DNA sequencing results. (B) Western blotting for ZEB1 shows ZEB1 protein levels in each of the clones. The predicted ZEB1 genotype as interpreted from the Western blot results are show in (A), with clones that did not show consistent predictions shaded in gray. Alpha-tubulin (TUBA) was used as a loading control. (C) Phase-contrast microscopy images showing cultures of each of the cell clones. (TIF) [file pone.0218279.s003.tif]

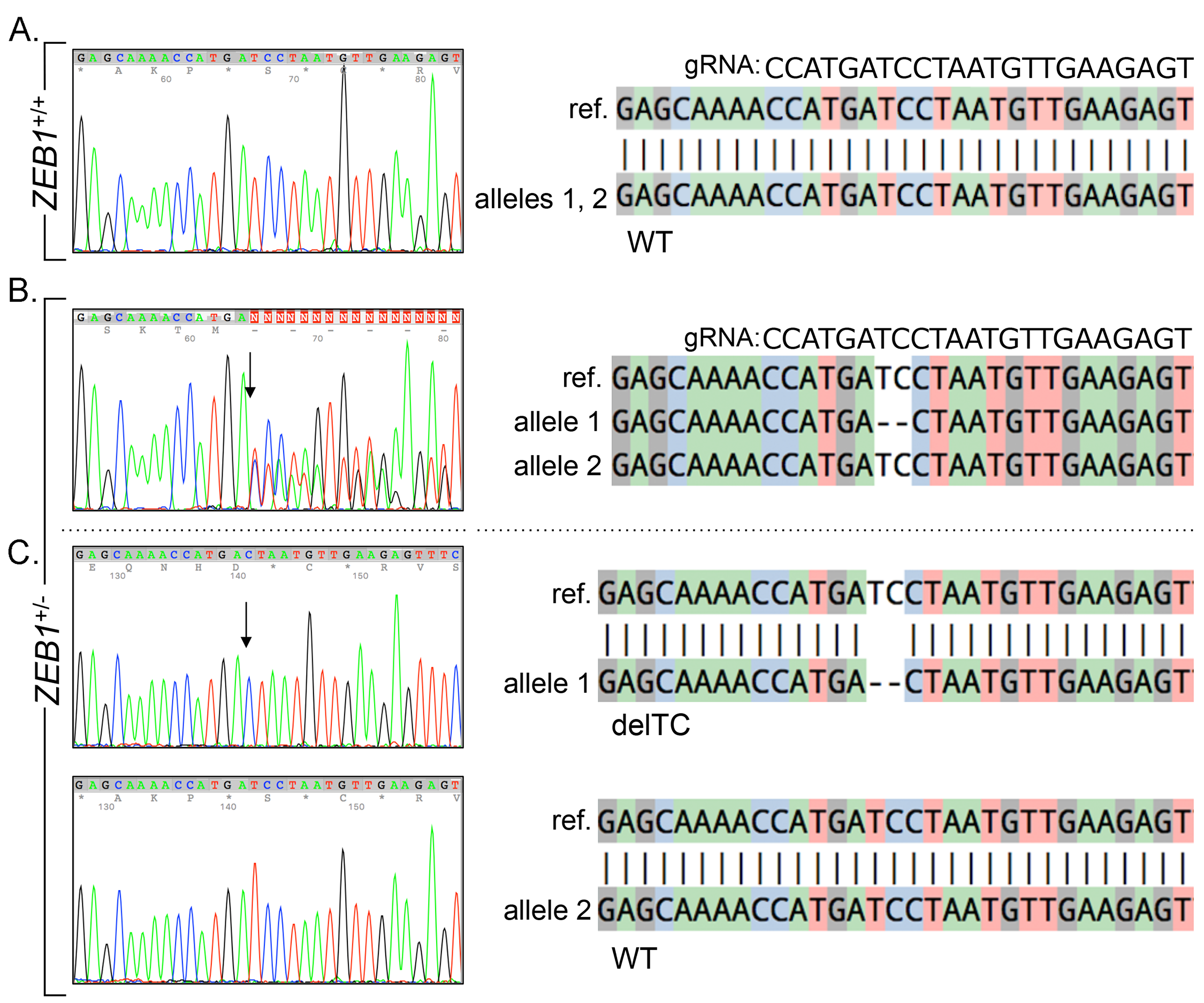

Supplement: S4 Fig — (A) Chromatogram shows Sanger sequencing results of ZEB1 exon 4 for the ZEB1+/+ CEnC line. Genomic DNA (diploid) template was used for sequencing. Sequence alignment using CRISP-ID was performed against a reference. Guide RNA sequence is shown above reference sequence. (B) Chromatogram shows sequencing results of ZEB1 exon 4 for the ZEB1+/- CEnC line. Genomic DNA (diploid) template was used for sequencing. Arrow indicates position of the introduction of an indel(s) by NHEJ repair. Sequence alignment to a reference sequence and allele prediction using CRISP-ID shows a mutant allele (delTC) and one wild type allele. (C) Independent sequencing of the individual alleles confirmed that the mutant allele harbors a deletion (delTC), while the second allele was wild type. Arrow indicates position of delTC. (TIF) [file pone.0218279.s004.tif]

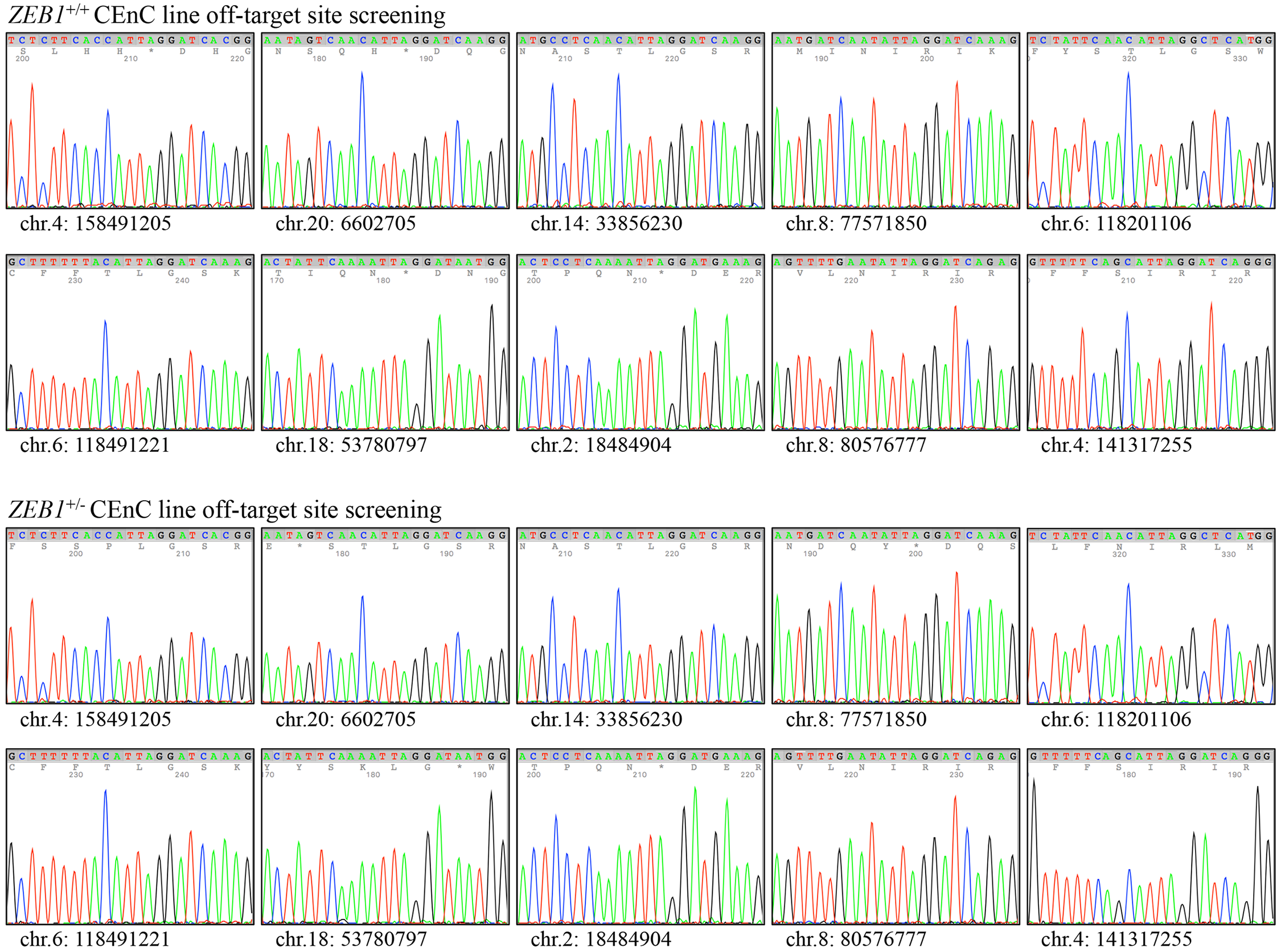

Supplement: S5 Fig — Chromatograms show sequencing results of the ZEB1+/+ (top set) and ZEB1+/- (bottom set) CEnC lines for the 10 off-target sites with the highest scores (see S2C Fig). Chromosome and position are listed under each chromatogram. Primers for off-target sequencing are in S1 Table. (TIF) [file pone.0218279.s005.tif]

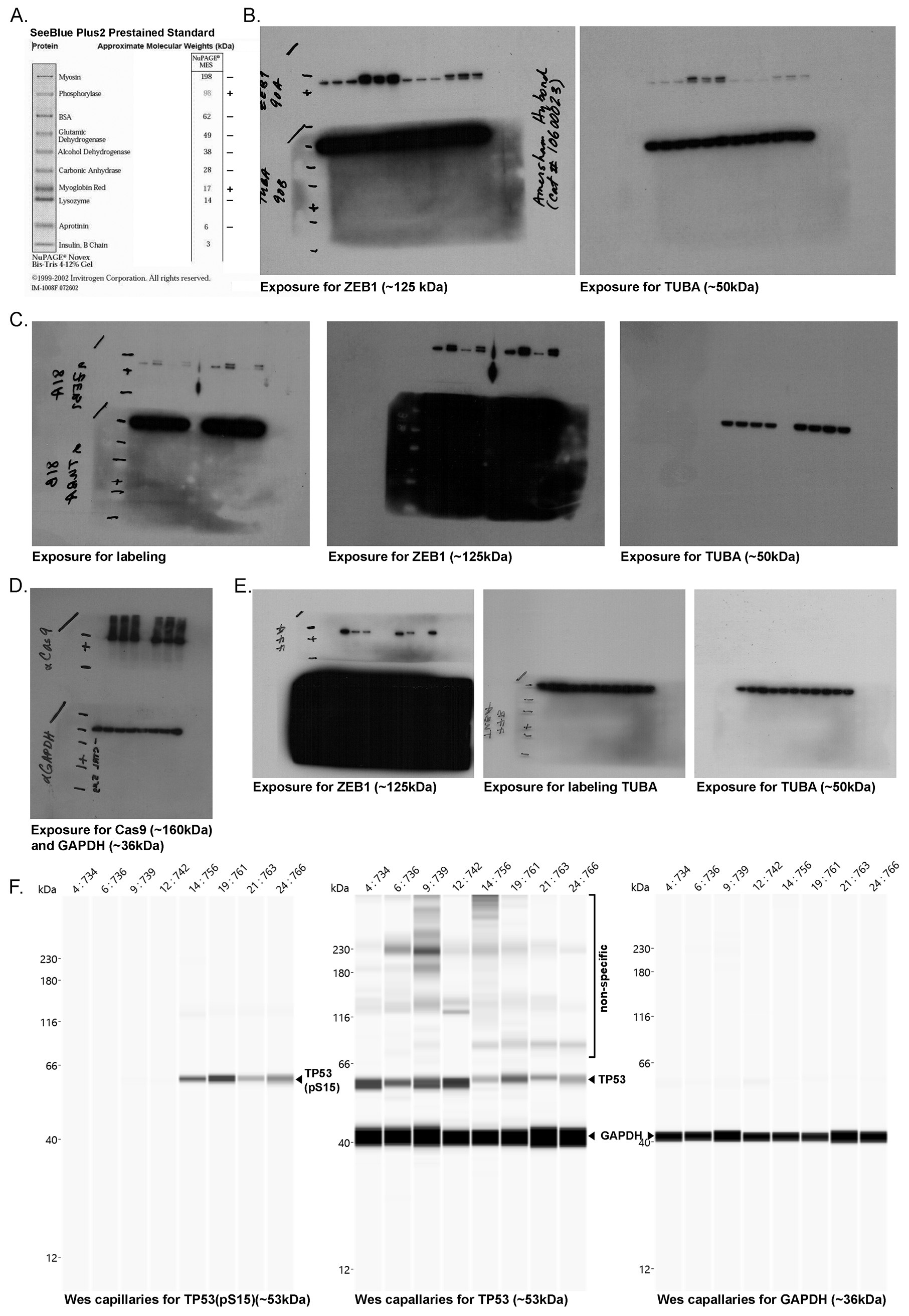

Supplement: S6 Fig — (A) The SeeBlue Plus2 prestained protein standard was used to determine protein sizes by Western blot. Dash and plus symbols correspond to the markings on the autoradiographs. (B) Western blot data for results shown in Fig 2C Western blot data for results shown in Fig 4. Two sets of the same samples were run on the same SDS-PAGE. (D) Western blot data for the results shown in S2 Fig. Two sets of the same samples were run on the same SDS-PAGE. (E) Western blot data for the results shown in S3F Fig. Western data obtained with an automated capillary-based system (Wes). Western data for results shown in Fig 7. The results for TP53 and GAPDH were obtained from a single multiplexed run using anti-TP53 and anti-GAPDH antibodies. The exposure showing both proteins was used for TP53 and the second exposure showing only a single protein was used for GAPDH. (TIF) [file pone.0218279.s006.tif]
