# Supplementary material for: ZEB1 insufficiency causes corneal endothelial cell state transition and altered cellular processing
Source: PLoS One. 2019 Jun 13;14(6):e0218279. doi: 10.1371/journal.pone.0218279 (PMC6564028; doi:10.1371/journal.pone.0218279)
Supplement: S1 Table — (DOCX) [file pone.0218279.s007.docx]

Table S1. PCR primers for sequencing CRISPR-Cas9 off-target sites.

| **Primer Sequences (5’-3’)** | **Amplicon Size (bp)** |
| --- | --- |
| F1-TGCTAGGATGCCACTAAGCTGT | 438 |
| R1-TGGGAGCAGCTCACTTCTCT |  |
| F2-ATGGGTTAGAGCAACCAGTGAATTA | 401 |
| R2-ACTGTAACCCTCTACTTCTGTAGGC |  |
| F3-ATGAGAATCAGGTGGGCGTCT | 438 |
| R3-CTGAGGGGCTGACAACACTGA |  |
| F4-TACAACCACTGAACCAGACCCTA | 402 |
| R4-CACTGACCCCAATGCTTCCA |  |
| F5-GTCTTCAGTTCTCTCTCTGAAGCA | 419 |
| R5-CGTTGGTCCATGAGCCAAGATG |  |
| F6-CTGTGCTCTATTCTGTGAGCCAAA | 464 |
| R6-TGTGCCTTAGAAGCAGTCCAACA |  |
| F7-AGGCTCATTTGGCGTGCTTT | 407 |
| R7-GCTCCCAGCCCTTTGTCCA |  |
| F8-GAGCAAAGGCCTTGTCCTATTCA | 401 |
| R8-ACATGCAAAAGTGTGCTCCCAA |  |
| F9-TGTCTGGTCCTTTGCGTACCAT | 416 |
| R9-AAGGGCCTTGACAAACAGGTCA |  |
| F10-CACACCCAATCCGACATGCTG | 411 |
| R10-GGCACAAACAAAAGGGAGGGAAA |  |

Note: annealing temperature used for PCR was 61°C for all primer pairs.
